# A guide for social science journal editors on easing into open science

**DOI:** 10.1186/s41073-023-00141-5

**Published:** 2024-02-16

**Authors:** Priya Silverstein, Colin Elman, Amanda Montoya, Barbara McGillivray, Charlotte R. Pennington, Chase H. Harrison, Crystal N. Steltenpohl, Jan Philipp Röer, Katherine S. Corker, Lisa M. Charron, Mahmoud Elsherif, Mario Malicki, Rachel Hayes-Harb, Sandra Grinschgl, Tess Neal, Thomas Rhys Evans, Veli-Matti Karhulahti, William L. D. Krenzer, Anabel Belaus, David Moreau, Debora I. Burin, Elizabeth Chin, Esther Plomp, Evan Mayo-Wilson, Jared Lyle, Jonathan M. Adler, Julia G. Bottesini, Katherine M. Lawson, Kathleen Schmidt, Kyrani Reneau, Lars Vilhuber, Ludo Waltman, Morton Ann Gernsbacher, Paul E. Plonski, Sakshi Ghai, Sean Grant, Thu-Mai Christian, William Ngiam, Moin Syed

**Affiliations:** 1 Department of Psychology, Ashland University, Ashland, USA; 2 Institute for Globally Distributed Open Research and Education, Preston, UK; 3 Maxwell School of Citizenship and Public Affairs, Syracuse University, Syracuse, USA; 4 Department of Psychology, University of California, Los Angeles, USA; 5 Department of Digital Humanities, King’s College London, London, UK; 6 School of Psychology, College of Health & Life Sciences, Aston University, Birmingham, UK; 7 Department of Government, Harvard University, Cambridge, USA; 8 Dartmouth Center for Program Design and Evaluation, Hanover, USA; 9 Department of Psychology and Psychotherapy, Witten/Herdecke University, Witten, Germany; 10 Department of Psychology, Grand Valley State University, Allendale, USA; 11 American Family Insurance Data Science Institute, University of Wisconsin-Madison, Madison, USA; 12 Nelson Institute for Environmental Studies, University of Wisconsin-Madison, Madison, USA; 13 Department of Psychology, University of Birmingham, Birmingham, UK; 14 Meta-Research Innovation Center at Stanford, Stanford University, Stanford, USA; 15 Stanford Program On Research Rigor and Reproducibility, Stanford University, Stanford, USA; 16 Department of Epidemiology and Population Health, Stanford University School of Medicine, Stanford, USA; 17 Department of Linguistics, University of Utah, Salt Lake City, USA; 18 Department of Psychology, University of Graz, Graz, Austria; 19 Department of Psychology, Iowa State University, Ames, USA; 20 School of Social & Behavioral Sciences, Arizona State University, Tempe, USA; 21 School of Human Sciences and Institute for Lifecourse Development, University of Greenwich, London, UK; 22 Department of Music, Art and Culture Studies, University of Jyväskylä, Jyväskylä, Finland; 23 Office of Scientific Integrity, Duke University, Durham, USA; 24 National Agency for Scientific and Technological Promotion, Córdoba, Argentina; 25 School of Psychology and Centre for Brain Research, University of Auckland, Auckland, New Zealand; 26 Facultad de Psicología, Universidad de Buenos Aires, Buenos Aires, Argentina; 27 CONICET, Buenos Aires, Argentina; 28 ArtCenter College of Design, Pasadena, USA; 29 Faculty of Applied Sciences, Delft University of Technology, Delft, Netherlands; 30 The, The Alan Turing Institute, Turing Way, London, UK; 31 Department of Epidemiology, UNC Gillings School of Global Public Health, Chapel Hill, USA; 32 Inter-University Consortium for Political and Social Research (ICPSR), University of Michigan, Ann Arbor, USA; 33 Olin College of Engineering, Needham, USA; 34 Department of Psychology, Rhodes College, Memphis, USA; 35 Economics Department, Cornell University, Ithaca, USA; 36 Centre for Science and Technology Studies, Leiden University, Leiden, Netherlands; 37 Department of Psychology, University of Wisconsin-Madison, Madison, USA; 38 Department of Psychology, Tufts University, Medford, USA; 39 Department of Psychology, University of Cambridge, Cambridge, USA; 40 HEDCO Institute for Evidence-Based Practice, College of Education, University of Oregon, Eugene, USA; 41 Odum Institute for Research in Social Science, University of North Carolina at Chapel Hill, Chapel Hill, USA; 42 Institute of Mind and Biology, University of Chicago, Chicago, USA; 43 Department of Psychology, University of Chicago, Chicago, USA; 44 Department of Psychology, University of Minnesota, Minneapolis, USA

**Keywords:** Open science, Journal editing, Scholarly publishing, Peer review

## Abstract

Journal editors have a large amount of power to advance open science in their respective fields by incentivising and mandating open policies and practices at their journals. The Data PASS Journal Editors Discussion Interface (JEDI, an online community for social science journal editors: www.dpjedi.org) has collated several resources on embedding open science in journal editing (www.dpjedi.org/resources). However, it can be overwhelming as an editor new to open science practices to know where to start. For this reason, we created a guide for journal editors on how to get started with open science. The guide outlines steps that editors can take to implement open policies and practices within their journal, and goes through the what, why, how, and worries of each policy and practice. This manuscript introduces and summarizes the guide (full guide: https://doi.org/10.31219/osf.io/hstcx).

## Background

Many research practices that were previously considered acceptable, or even normative, in the social sciences are now widely recognized to work against our collective goal of establishing a cumulative knowledge base rooted in rigorous evidence. Issues with credibility have been documented across different disciplines [1–7], and there is increasing awareness that many scientific incentives actively encourage, reward and propagate poor research and statistical methods [8, 9]. Indeed, existing scholarly practices have strong roots in a deeply embedded problematic research culture that favors quantity over quality of research products, “positive” results, and flashy findings, all occurring within a hierarchical, status-based system [10].

To overcome many of these issues, open science (also referred to as “open research” or “open scholarship”) has been advanced as an alternative model for science and one that will better contribute to our collective goals and build a more reproducible scientific knowledge base. Yet, knowledge of open science principles and practices remains uneven across different social scientific constituencies. The purpose of the present article is to focus on a particularly important constituency – journal editors – by providing a guide that will help them to adopt open science practices in their journals.

Open science is a broad term that does not have a single agreed upon definition, with different definitions foregrounding different aspects of the scientific ecosystem. For example, the UNESCO Recommendation on Open Science [11] adopts a broad definition that highlights the system of knowledge production:

*“…open science is defined as an inclusive construct that combines various movements and practices aiming to make multilingual scientific knowledge openly available, accessible and reusable for everyone, to increase scientific collaborations and sharing of information for the benefits of science and society, and to open the processes of scientific knowledge creation, evaluation and communication to societal actors beyond the traditional scientific community. It comprises all scientific disciplines and aspects of scholarly practices, including basic and applied sciences, natural and social sciences and the humanities, and it builds on the following key pillars: open scientific knowledge, open science infrastructures, science communication, open engagement of societal actors and open dialogue with other knowledge systems.” (*https://unesdoc.unesco.org/ark:/48223/pf0000379949*)*

In contrast, the Framework for Open and Reproducible Research Training [12] defines open science more narrowly toward specific behaviors of researchers:

*“An umbrella term reflecting the idea that scientific knowledge of all kinds, where appropriate, should be openly accessible, transparent, rigorous, reproducible, replicable, accumulative, and inclusive, all which are considered fundamental features of the scientific endeavor.”* [13].

Underlying these definitional differences are shared values in the conduct and dissemination of science, and the need to move toward the principles and behaviors of open science has been widely recognized across the sciences. Research communities across many disciplines have begun to develop stronger norms inspired by open science, including psychology [2, 14–16], genetics [17], biomedicine [18], animal behavior [4, 19], economics [20–24], education [21, 25–29], political science [30], public health [31, 32], science and technology studies [33], scientometrics [34], and sociology [35, 36], among others (see ([37]). Despite some progress, all stakeholders in the system need to do better at adopting and implementing open science practices, and our focus is on how to help editors accomplish this.

Over the last two decades, the operational procedures of scholarly social science have been substantially modified to facilitate the goals of open science [10, 14, 38]. However, the shift toward open science remains a work-in-progress. Recognizing that open science is fundamentally about behaviors, various established theories of behavior change, including the Behaviour Change Wheel [39] and Theoretical Domains Framework [40], have been applied to understand how to increase uptake of open science among researchers [41, 42] and journal editors [43]. It is clear that multiple institutional stakeholders – funders, disciplinary associations, data repositories, universities, publishers, preprint servers, and journals – have the capacity to influence how research is conducted by enacting one or more of these strategies [44, 45].

Among the different stakeholders, journals are in a strong position to foster open science practices. They are particularly influential institutions in the academic ecosystem because they are a major vehicle for organizing and disseminating academic communications, promoting knowledge, and producing success signals for individual researchers [46]. This influence has not always been beneficial to science, as journal policies and practices are one source of a problem that open science is meant to address (for example publication bias), but it is precisely this capacity to incentivize and shape scholarly behavior which now offers a broad opportunity to promote transparency and openness.

The degree of power that journal editors have to enact change in policies varies considerably across journals, as publishers and scientific societies often play central roles in setting policies. Nevertheless, journal editors are in a position to be a major influence on policies that can help move disciplines toward more rigorous open science and improve research culture. Most obviously, journals’ *mandates* to authors can make publication conditional on following open science practices [14]. Less directly, journals can include processes that *endorse, encourage,* and *reward* open science, such as promoting replication, offering the Registered Reports publishing model, and encouraging preprinting. Journals that instantiate open science can also be *opinion leaders* in their respective disciplines, helping to make the practices more visible and customary.

With this potential in mind, the Transparency and Openness Promotion (TOP) Guidelines [14] were developed to provide tools (including template policy text) to help journal editors adopt open science policies within their journals. The TOP Guidelines are a resource for editors, covering many areas of open science (data citation; data, materials, and code transparency; design and analysis; preregistration; replication) currently available in English, Spanish, Portuguese, and Finnish (https://www.cos.io/initiatives/top-guidelines). In a separate but related initiative, the Center for Open Science (COS) ranks journals on their adherence to these guidelines via the TOP Factor, a metric that has been proposed as an alternative to citation-based metrics such as the Journal Impact Factor [47, 48].

Whereas the TOP Guidelines and TOP Factor provide a good deal of information for and about journals, there are at least two gaps that the current paper hopes to fill. First, TOP covers a very limited set of behaviors that, while useful, do not cover the full spectrum of open science practices (for example topics related to open and transparent peer review, open access, and encouraging diversity). Second, although the TOP Guidelines provide information on the different standards (the *what,* including template policy text), they do not focus on *why* editors should implement these standards, *how* editors should implement the procedures and practices that uphold the policies, and the *worries* (and associated mitigations) they may have about implementing these new procedures and practices. Some recent work has begun to explore these questions (for example [43, 49], but this work has been focused on the limited scope of the TOP Guidelines. Thus, our focus in the present guide is to expand the range of open science considerations for journal editors to the broad spectrum of issues that may be relevant across the social sciences, while still maintaining a connection to TOP where relevant.

**Fig. 1:**
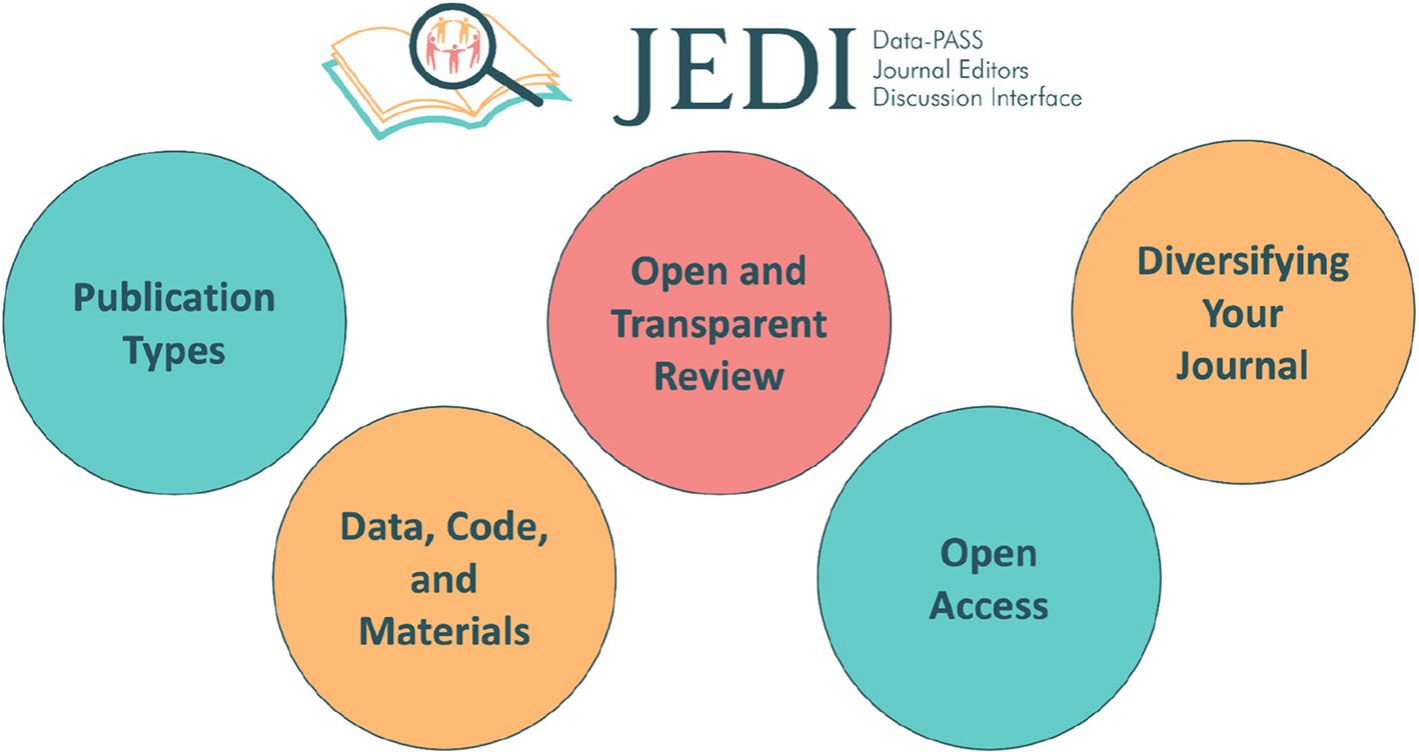
Key topics covered in the full guide

Accordingly, the purpose of the present guide is to help editors “ease into open science” by providing information on the *what*, *why*, *how*, and *worries* associated with adopting a broad range of open science initiatives at their journals (see an example for Registered Reports in Table 1 and a list of key topics covered in Fig. 1). This approach was modeled on Kathawalla et al.’s guide [50] for graduate students and their advisors. We hope that the present article will prove similarly useful for editors given their pivotal role in the scientific ecosystem. Editors are typically overburdened with multiple roles and obligations, including responsibilities as researchers, teachers, managers, and members of their individual scientific communities. Indeed, editorial positions are typically taken on in addition to other “regular” work, often for little or no compensation. Thus, this guide is especially well-catered to the majority of editors who have limited time to dedicate to editing, and even less time for designing and implementing new journal policies and practices.

**Table 1:** An example table of one of 37 policy/practices from the full guide

Policy/Practice:	**Publish Registered Reports**
What:	Registered Reports (RRs) are “a scientific publishing format that includes an initial round of peer review of the background and methods (study design, measurement, and analysis plan); sufficiently high quality manuscripts are accepted for in-principle acceptance (IPA) at this stage…Following data analyses and write up of results and discussion sections, the stage 2 review assesses whether authors sufficiently followed their study plan and reported deviations from it (and remains indifferent to the results).” [13] RRs have two main features: 1) peer review before data collection, and 2) acceptance regardless of the results obtained [51]
Why:	The RR format redirects the review's focus toward the proposed research question and methodology, rather than the anticipated results of the study [13] The RR format eliminates several questionable research practices, such as low statistical power, selective reporting of results, and publication bias, while providing the flexibility to report any unexpected findings (https://www.cos.io/initiatives/registered-reports) Because RR reviewers evaluate a Stage 1 study proposal and then evaluate the final Stage 2 manuscript, any deviations can be spotted clearly and reported in a more transparent way. Instead, with study preregistration, discrepancies between the preregistration and the final article may be harder to spot [52] With Registered Reports reviews have added value, as the feedback and suggestions can still be incorporated into the study, rather than addressed afterwards
How:	The Center for Open Science provides resources for editors (see “Resources for Editors” and “FAQ” tabs https://www.cos.io/initiatives/registered-reports). This includes email templates for all key sections, submission templates and journal policy guidelines
Worries:	RRs are not necessary or relevant for my discipline • RRs can be conducted in any field that follows a research workflow which begins with study planning and design • RRs are especially helpful in any discipline where publication bias and questionable research practices exist We work on rapid/fast-paced science for immediate impact – RRs are too slow • RRs can be achieved in a short time scale. Journals can offer 'rapid response' RRs for time-sensitive projects (for example research in response to the COVID pandemic [53] • Research that is fast-paced may lead to more errors, and so the RR format ensures that the study design can be reviewed before data collection commences to reduce the likelihood of errors at the design stage I only want to publish significant results at my journal, because these are the results that will be cited more • A well designed study should lead to informative results regardless of the outcome • The increase of “null” results in RRs [54] may be a better representation of the research being conducted as a whole • RRs are cited equivalently to, or at a slightly higher rate than, ‘traditional’ articles [55] • RRs are judged (through masked peer-review) as being higher in rigor, quality, and detail, as well as comparable in creativity and importance [56] RRs create more administrative burden, due to reviewers being required at Stage 1 and Stage 2 • Compared to a ‘traditional’ manuscript, the RR manuscript is split into two stages: *Stage 1*, which focuses on the Introduction, Methods, and Analysis Plan, and *Stage 2*, which focuses on the Results and Discussion [57]. The *amount of total article* to be reviewed for an RR is therefore the same as a traditional manuscript, but of course the burden on reviewers and editors can be larger (especially because of the need to compare the Stage 1 and Stage 2 reports). As with everything else in this guide, editors will have to weigh up the pros and cons of adding additional steps to the review process It will be harder to find reviewers • There is no evidence that this is the case, but you can outsource peer review to PCI-RR if you’re worried about this (see next box in full guide) • Most RR reviewers are more motivated because their feedback can directly impact the work at a crucial time (before data collection) I might lose the reviewer of a Stage 1 manuscript at Stage 2 • While peer-reviewers are invited to be reviewers for both stages of the RR review process, there may sometimes be difficulties in retaining reviewers for both the Stage 1 and Stage 2 reviews. In this case, a new reviewer should be sought at Stage 2. This is not too different from a revision of a traditional article format except that the Stage 2 reviewer can still refer back to the Stage 1 submission RRs are not a suitable format for qualitative research • RRs are not just for quantitative research; they are also suitable also for qualitative studies. For authors, being able to receive feedback and transparently work toward agreed research questions can be valuable. For editors, being able to ensure the quality of the research plan and data/materials sharing can increase the quality of the publications Authors may not want to use the RR format • Adding Registered Reports as an option does not require authors to use it, they can simply continue to submit traditional reports if they so choose. It does not require an extra load of resources to implement, so it is not a problem even if it is seldomly used • Most journals offering the RR format also offer the “‘traditional” publishing track The process of adding RRs to a journal is complicated and arduous • Installing RRs has become increasingly easy over time. With around 300 journals now offering them, all major publishers have at least one adopter under their umbrella. In many cases, the format and workflow can be imported very easily between journals and all central resources/templates required are openly available (see “How” section). The increased frequency of adoption means that major publishers are generally familiar with how to implement them in the manuscript systems
Resources:	COS Registered Reports: https://www.cos.io/initiatives/registered-reports [58]: Registered Reports: A Method to Increase the Credibility of Published Results [57]: The past, present and future of Registered Reports [51]: Opening the Door to Registered Reports: Census of Journals Publishing Registered Reports (2013–2020) [59]: Registered reports for qualitative research Registered Report Census Database: https://datastudio.google.com/u/0/reporting/95aff8ff-7ec6-4363-bf05-f81f61215bd3/page/shIqB

We intend for the present guide to be useful in at least two ways. First, it provides straightforward descriptions of open science policies, procedures, and practices, as well as guidance and signposted resources for how to implement them, with consideration of their potential challenges and costs. When making recommendations, we rely on findings from science studies research where they are available, but not all recommendations are evidence-based. Instead, some of them draw upon our own experiences as authors, peer reviewers, and journal editors. We hope that this guide will help to encourage future empirical studies on the effects of different policy changes (for example randomized controlled trials) where they have not yet been conducted.

Second, we reject an “all or nothing” approach to open science. Different journals will have different needs, resources, audiences, governance structures, and any number of other factors that will determine which open science practices they do or do not want to adopt. The current guide is designed so that editors can follow a “buffet approach” to implementing open science initiatives [60], whereby editors can pick and choose whatever makes sense for their journal, resources, and field. The number of possible reforms is large and can potentially feel overwhelming, and so we stress the need to “ease in” and adopt reforms as feasible.

### Guide development

This guide emerged collaboratively, led by leadership from the Journal Editors Discussion Interface (JEDI; https://dpjedi.org), a Data Preservation Alliance for the Social Science (Data-PASS; http://www.data-pass.org) initiative. Data-PASS is a voluntary partnership of organizations created to archive, catalog, and preserve data used for social science research. Data-PASS was initially formed with the goal of its members providing a shared catalog of holdings, and serving as alternative venues should a member be unable to continue preserving data. Over time, Data-PASS’s members have also collaboratively developed and implemented additional supporting resources for open science. For example, between 2016 and 2020, Data-PASS held a series of workshops, bringing together social science journal editors and representatives from Data-PASS to discuss issues surrounding open science in journal editing. In 2021, NSF funding facilitated the launch of JEDI – an online forum where social science journal editors can ask and answer questions, share information and expertise, and build a fund of collective knowledge. JEDI is composed of a Google group of several hundred members that functions as a listserv and a collection of resources (https://dpjedi.org/resources) compiled from conversations in the group.

While discussion of any editorial function or concern is encouraged, a large focus of conversations on the listserv (and therefore also the resources collection) has been on journal open science initiatives. In May 2022, we held a workshop focused on open science and the future of scholarly publishing that had over 100 registrants (“*May the force be with you: Resources to help journal editors advance their fields*”: https://dpjedi.org/events/may-the-force-be-with-you). This workshop resulted in many valuable additions to our resource collection. However, due to the bottom-up nature of how the resources had been collected, there was large variation in the amount, quality, and type of content for different topics. Therefore, a gap was identified for a comprehensive guide for social science journal editors on open science initiatives. This guide was originally conceptualized by a small team from JEDI leadership: Priya Silverstein (previous JEDI community manager), Moin Syed (past JEDI steering committee member), and Colin Elman (PI on the NSF grant supporting JEDI, and ex officio steering committee member).

A first draft of this guide was written by Priya Silverstein. This draft formed the starting point for a hackathon (a type of goal-focused participatory workshop; [61]) at the 2022 annual meeting of the Society for the Improvement of Psychological Science (SIPS) where approximately 20 contributors came together to draft around 30 sections, covering a wide range of journal open science initiatives. This was a largely psychology-focused team, and so following the hackathon, the guide was opened up for contributions from the JEDI steering committee. The JEDI steering committee is composed of 13 invited members: six representatives from the data repositories included in Data-PASS and one editor each from anthropology, criminology, economics, education, political science, psychology, and sociology. After the JEDI steering committee contributed to the guide, it was opened up for contributions from the wider JEDI community of over 400 members, including editors from across the social sciences and “Scholarly Knowledge Builders” (JEDI topic experts in different aspects of open science, metascience of peer review, and publishing). Priya Silverstein took the lead in integrating contributions, comments, and edits until a long-form guide had been finalized (see [62] for the full guide). The current paper serves as a shortened summary of the full guide, including the initiatives that we believe are relatively easy to implement and/or likely to apply to most (or many) social science journals. JEDI has now received further funding and, as part of its expansion and continuation, the full guide will continue to be expanded and updated regularly.

### Summary of the guide for social science journal editors

In this summary, we have grouped the initiatives included in the full guide into three categories: those relating to the principles of Transparency, Credibility, and Accessibility. These are not to be taken as rigid categories. Rather, this categorization scheme is to emphasize that within open science there are different goals that can be achieved through different initiatives. Many of the initiatives will work towards more than one of the principles, as well as other principles that we do not specifically emphasize here (for example Reproducibility). Moreover, some of the initiatives have the potential to have differential impact on the principles, and thus could possibly be in conflict with one another (for example open peer review should increase transparency, but could reduce accessibility if some authors or reviewers are reluctant to engage in the practice). In what follows, we briefly review each of the three principles, and highlight select entries from the full guide to provide further explanations of the initiatives, the benefits of adopting them, the potential concerns that might arise, and their mitigations. Each **emboldened** initiative has its own dedicated table in the full guide.

#### Transparency

The principle of transparency pertains to researchers being honest and forthcoming about all aspects of the scientific process [13]. For researchers, transparency involves disclosing all theoretical, methodological, and analytic decisions made throughout the research cycle. Similarly, transparency for journals and editors involves documentation and availability of all phases of the publication cycle. Behaviors in support of transparency are meant to reduce the knowledge asymmetry between producers and consumers of research, allowing the latter to make more informed judgments about research quality [63]. Journal editors are well-positioned to advance initiatives that support transparency among researchers and at the journals themselves. The full guide details several specific initiatives, but here we briefly summarize a small number that have seen widespread adoption and are relatively straightforward to adopt.

First, journals can encourage or mandate that authors **Share Data** (either alongside an empirical manuscript or through publishing a **Data Descriptor**), **Share Code**, **Share Materials** associated with their study (i.e. the different measures, stimuli, and procedures that were used to produce the findings reported in the article), and/or require **Adherence to Methodological Reporting Guidelines** (using transparency standards to specify specific elements of study design which should be disclosed). These initiatives allow interested parties to reproduce the study findings, reuse research components, fit alternative models, catch errors, and critically evaluate research decisions and outcomes more rigorously.

There are many different options for journals to facilitate authors’ sharing data, code, and materials, ranging from informal incentives to formal mandates. For example, journals can offer open data and open materials badges as a way of incentivising sharing (although note that the efficacy of badges is still debated, see for example [64]). However, studies have found that data indicated as “being available upon request” rarely are actually available in practice [65]. Journals could instead implement policies to make acceptance conditional upon direct links to open data, code, and materials (although, note, even having policies to mandate data and code sharing do not mean that analyses are reproducible [66]). Journals can then decide whether they will check whether the shared materials are complete or not (is this the responsibility of the author or of the journal). For editors, it is obviously more resource intensive to check whether materials are complete, so this step is only possible if the journal has the personnel, time, and money to do this. Alternatively, the guidelines to authors can include information about the sharing of such research components and clear instructions that outline their responsibility to ensure these are complete.

Sharing data, code, and materials is a useful first step for promoting transparency, but even if fully available, researchers could have engaged in undisclosed data-dependent decision-making (for example questionable research practices such as *p*-hacking), and thus full transparency is still not realized. **Registration**, sometimes also called preregistration (see [67]) can bolster transparency and involves creating an accessible, time-stamped plan that details the study research questions and/or hypotheses, design, and analysis. Journals can incentivise preregistration through offering a preregistration badge to articles that meet set criteria [68]. Journals can also require a statement specifying whether or not a study has been preregistered, require that, if a study was preregistered, the preregistration protocol is available for peer review, or require preregistration for all empirical work. Editors may worry that preregistration is not relevant to their particular field or methodology, but there are now options for preregistration across a wide variety of disciplines and types of research (for example exploratory and confirmatory research, quantitative and qualitative; [69, 70], though there may be variations in how preregistration is used within different epistemologies.

Taking this practice a step further, journals can adopt the **Registered Reports** (RR) publishing format [57]. RRs are a publishing format where initial peer review is performed on a study protocol before data collection and/or analyses are conducted. Accepted Stage 1 manuscripts are given ‘In-Principle Acceptance’ (IPA), moving the focus to the process of research and away from the results [13, 71]. The Center for Open Science provides resources for editors wanting to adopt RRs in their journal (see “Resources for Editors” and “FAQ” tabs on the Registered Reports page: https://www.cos.io/initiatives/registered-reports), and more than 300 journals currently offer this publishing format. These resources include email templates for all key sections, submission templates, and journal policy guidelines. Editors may worry that RRs are not necessary or relevant to their discipline, but they can be conducted in any field that follows a research workflow which begins with study planning and design. Journals can also participate in **Peer Community In Registered Reports** (PCI-RR), which offers free and transparent pre- and post-study recommendations by overseeing the peer review of RR preprints (see https://rr.peercommunityin.org/). PCI-RR accepts submissions proposing either the analysis of new data or pre-existing data using levels of bias control, and is therefore a suitable format for studies at any stage of the research process. The peer review is conducted independently of journals by expert ‘recommenders’ and this is endorsed by a growing list of journals that accept PCI-RR recommendations (known as ‘PCI-RR friendly’ journals). So, journal editors can outsource part, or all, of the peer review process.

Turning to journal-focused rather than author-focused initiatives that support transparency, journals can adopt **Transparent Peer Review**, in which they make the peer review reports, editorial decisions [72], and author reply letters openly available alongside published manuscripts. Instituting transparent peer review will require that the online publishing platform allows this logistically, or the materials can be uploaded as supplementary materials. Sometimes a distinction is made between transparent peer review, where the content of the review process is made open, and **Open Peer Review**, where the reviewers’ identities are made open as well. Open peer review comes with additional concerns; for example, some people are concerned that reviewers will be treated unfairly for giving unfavorable reviews or that open identities will enable bias and retaliation. For a balanced scoping review of the pros and cons of open peer review, see [73].

#### Credibility

The principle of credibility refers to the degree of trustworthiness or believability of research findings. Whereas transparency focuses on making the research process and products *open* to evaluation, credibility relates to the evaluations of these processes and products for their quality. In many ways transparency is a necessary, but not sufficient, condition for credibility [74]. That is, sharing data and materials, or preregistering a study, do not enhance credibility of findings on their own, but allow for a fuller assessment of it. Behaviors in support of the principle of credibility are aimed at increasing the trustworthiness of a study, or body of research. Here, we highlight three initiatives aimed at encouraging the credibility of published research.

First, journals can explicitly encourage the submission of **Replication Studies**. Replication studies are a broad class of studies that can vary in motivation and procedure, but generally refer to a study for which any outcome would be considered diagnostic evidence about a claim from prior research [75]. Publishing replication studies enhances credibility because they are informative about how well previously observed results hold up in new and different settings. Although researchers have to *conduct* replications in order for journals to *publish* them, journals have a long and notorious history of discouraging the submission of replication studies in favor of novel findings [76, 77]. Instead, journals can make explicit that they encourage replications through clear language in their guidelines, and/or through implementing “the pottery barn rule” [78], whereby journals agree to publish a direct replication of any study previously published in their journal.

A particularly useful format for receiving replication studies is via the aforementioned **Registered Reports** publishing format. From the journal side, Registered Reports promote credibility because they signal that the journal evaluates and selects articles for publication based on their conceptualization and methods, and not based on the perceived novelty or potential impact of the findings. This format is well-suited to replication studies because it requires clear statements of the criteria that will substantiate a claim of replication *before* the results are known, thus combatting interpretative bias. Another solution to combatting interpretive bias is to have **Results Masked Review**, where the replication has been completed but only the introduction and methods are initially reviewed. Results masked review can be an especially useful route for publishing replication studies that have been completed but previously file-drawered.

Replication studies are seen as one important, even if currently limited, behavior that can increase the cumulativeness of scientific findings, contributing to the “self-correcting” nature of science. Contrary to the meaning inherent in the term “self-correcting,” science does not, in fact, correct itself [79], and is instead “other-correcting” [71]. People must actively work to correct the scientific record, and in this regard, journal editors can play a key role. It is incumbent on editors to act swiftly and prudently when **Handling Corrections** (updating an article to correct something and/or publishing an erratum or corrigendum) and **Retractions** (indicating that previously-published articles should no longer be relied upon). Retraction best practice can include outlining the specific reasons and timeline/history of retractions, and ensuring that there is a link to an open access version of retraction information on the manuscript webpage [80]. Beyond those behaviors that are in response to problems that arise, journals can commit to **Publishing Scientific Critique** [81], which involves publishing peer-initiated critical discourse related to specific research articles previously published in the same journal. The decision of whether to go for a retraction, correction, or publishing scientific critique can be made based on how serious the issue is with the original article (note that this can be difficult to determine in practice as people will disagree on how serious the issue is). Corrections should be reserved for changes that do not unequivocally undermine the findings of the original article (for example the labels of two groups on a graph have been swapped by mistake, but the conclusions still stand), whereas retractions should be reserved for changes that do (for example, the labels of two groups on a graph have been swapped by mistake, but the conclusions were based on the incorrectly labeled graph and so the data actually support the opposite conclusion). Post-publication critique can either stand alone (if there is room for nuance and disagreement), or can be accompanied by a correction or retraction (if the authors of the critique have found an error in the original manuscript). In the eventuality that an article is also retracted, the post-publication critique can still be published to aid transparency and document the article's history.

#### Accessibility

The principle of accessibility pertains to ensuring that all who are interested are able to consume, evaluate, and otherwise interact with research products and processes. Much of the discussion around accessibility in scientific publishing focuses on **Open Access**, which refers to articles being made freely available and reusable. Open access can take many forms, including Green Open Access (when the work is openly accessible from a public repository), Gold Open Access (when the work is immediately openly accessible upon publication via a journal website), and Platinum or Diamond Open Access (a subset of Gold OA in which all works in the journal are immediately accessible after publication from the journal website without the authors needing to pay an article processing charge [APC]) (Parsons et al., 2022). The emphasis on open access intersects with the desire to be inclusive to a diverse range of people, especially those from under resourced groups.

A strong initiative that both journal editors and authors can take to facilitate accessibility is through the integration of **Preprints**, which is a broad term that refers to versions of manuscripts posted to publicly-available repositories (e.g., arXiv, bioRxiv, PsyArXiv, SocArXiv). The term “preprints” applies to papers that have not (yet) been submitted for publication in a journal or are currently under review at a journal. “Preprints” can also be used to describe author-formatted versions of articles already published in a journal [82]. This latter category is more aptly labeled as “postprints,” yet are commonly referred to as preprints nevertheless. Even if a journal does not provide their own open access option, allowing authors to post preprints ensures that the research is accessible by everyone. Journals should allow authors to post the final accepted version to allow readers access to the most up-to-date version of a manuscript.

A behavior that is more directly in line with the inclusivity aspect of accessibility is **Supporting Language Editing**, which involves checking and correcting papers’ grammar, spelling, usage, and punctuation to ensure the meaning is understood by the intended audience. The ability to report on scientific findings via publication should not be overly inhibited due to reasonable limitations in written expression. For example, publishing in English language journals can be a barrier for researchers for whom English is not a first language, and supporting language editing is a way to support researchers if/when they write manuscripts in English. One way of implementing this is that existing publishing fees can be made to include editing services [83].

Finally, diversifying the journal editorial team can increase accessibility by providing leadership roles to scholars from under-represented backgrounds and countries. Diversifying the journal editorial team can be accomplished in at least two ways. An easy, low-effort approach is to issue an **Open Call for New Reviewers**, in which the journal makes clear that they seek to diversify the pool of reviewers that the editorial team relies on. This approach can be extended to search for new members of the editorial team as well, rather than relying on pre-existing networks that are prone to bias. These calls can be as simple as an open form linked on the journal’s website and social media accounts (if available). However, the process to become an editor at a journal is not transparent, which can be even more of a barrier for those people who come from historically excluded groups or from academic environments that contain fewer current editors, as they will not have access to this “hidden curriculum”. A more intensive approach is to develop a program focused on **Editorial Fellowships/Training**, which involves helping to train new associate/action editors from under-represented backgrounds. For example, the *American Psychological Association* now offers editorial fellowships at several of their journals, whereby fellows act as action/associate editors for a number of manuscripts over the year, with regular mentorship from a more experienced editor and financial compensation for their time.

### Considerations for implementing open science initiatives

The purpose of the present guide is to help editors implement a broad spectrum of open science initiatives at their journals. In the full guide, we provide straightforward descriptions of open science policies, procedures, and practices. We also detail *what* the initiative involves, *why* it should be implemented, *how* it can be implemented, and potential *worries* that could arise. We urge editors consulting this guide to endorse the “buffet approach” to open science [60] and not try to do too much at once, but rather to pick and choose the initiatives that make the most sense for the journal and the field. In the present paper we have highlighted a few of the key initiatives that editors could adopt, but the full guide includes over twenty other open science initiatives. Of course, even the full guide is not exhaustive; other actions by editors may increase openness and transparency at their journals. Moreover, as more metascientific research is conducted, we hope that we will also be able to better evaluate the effectiveness of many of these recommendations, especially across journals with different emphases (methodological, disciplinary, etc.).

We acknowledge that there are potential pitfalls surrounding some of the initiatives that we propose, which is why we felt it important to include a discussion of “worries” associated with each initiative in the full guide. For example, it is important to acknowledge that a more *open* science is not always a more *equitable* science [84–89]. In particular, it is possible that peer review with open identities (where the identities of reviewers are disclosed) could lead to reviewers being treated unfairly for giving unfavorable reviews, or to opening up the potential for bias or retaliation [90–92]. Openness is a value, but we have several other scientific values including but not limited to equity, diversity, speed, and cost-effectiveness. Editors will often have to make tradeoffs when deciding which initiatives to implement, in line with the values of their journal, scientific society (if associated with a society), and field [93].

Some of the worries we discuss are field or methodology specific. Particularly, social science employs a variety of methodologies which are often divided into “quantitative” and “qualitative.” It is important to remember, however, that these umbrella groups are largely labels of convenience, and that each, in turn, involves a range of approaches to data generation and analysis. In quantitative social science, for example, articles based on the statistical analysis of administrative data are likely to present different open science challenges than field experiments. Similarly, qualitative social science might involve a range of methods and epistemic commitments, such as ethnography, ordinary language Boolean process tracing, and Qualitative Comparative Analysis (QCA), which differ on data generation, the role of algorithmic analysis, and whether data are presented in tabular or textual form [94–96].

While open science needs to acknowledge and accommodate this heterogeneity, it also offers opportunities for different communities to learn from each other [97]. For example, while preregistration was pioneered in experimental research, in some circumstances qualitative researchers might benefit from its use [98, 99]. Similarly, positionality statements – statements used to contextualize the researcher and research environment [13, 100] – have been common in some varieties of qualitative research for several years, but are only just beginning to be considered a useful tool for quantitative research [101]. We also note that there are areas where there is ongoing debate about the possibility or usefulness of adopting certain open science initiatives for qualitative research. For example, some argue that replication should be encouraged in qualitative research [102], whereas others argue that there are still open questions about whether replication is possible, desirable, or even aligned with the epistemic foundations of qualitative research [85, 103]. Regardless of the perceived epistemic value of replications [85, 104], we believe it is non-controversial to suggest that journals should be open to publishing them, and surely should not have a policy that explicitly disallows them.

There is also debate about the advantages and disadvantages of open qualitative data, including the ethical considerations in making such data discoverable [105–107]. Although there is much to consider, qualitative data should not be automatically excluded from open data requirements [59]. While there will be some cases where data cannot or should not be shared (due to regulatory constraints imposed by law, the specifics of IRB approval, or ethical concerns), even sensitive data can sometimes be shared [108, 109] specifically with a reviewer or data editor solely for the purposes of review and who agrees to not make the data more widely available. Much “restricted secondary data” can be accessed by others, just not without restriction, and this can be clearly stated in a Data Availability Statement. The Qualitative Data Repository (https://qdr.syr.edu/) has many resources regarding qualitative data sharing, including how to manage access as necessary, which helps to balance participant privacy and researcher access. Note, issues around sensitive and restricted data sharing can also apply to quantitative data.

As an editor it is important to find the balance between adopting “easy” open science initiatives and thinking critically about whether and how these initiatives apply to your journal's particular field and/or methodologies. Guidelines need to “clearly articulate the kinds of research to which they apply” [97]. It can also be helpful to highlight initiatives that your journal has actively decided *not* to adopt, along with the reasons for this decision, for full transparency. Stakeholder engagement around proposed initiatives is important, especially if editors are worried about the reception or that they may be inadvertently burdening certain types of authors [45, 110].

More generally, we recognize that many factors will affect the logistics of adopting different initiatives. In particular, the administrative structure of a journal will be paramount in determining capacity for open science initiatives. Journal staffing may comprise any combination of editor-in-chief, associate editor(s), managing editor, or editorial assistant(s), and even roles like data editor, methodological transparency editors (see https://www.sree.org/research-transparency), and open science advisors. Individuals in these roles will have varying levels of the necessary experience, education, capabilities, expertise, and time allocated to create and implement open science initiatives. When developing new open science initiatives, editors should consider whether and how the existing administrative structure of their journal can support their implementation. They may also consider providing training and professional development for current staff, hiring new staff with appropriate qualifications, and/or creating opportunities for interested individuals to contribute (for example positions on an open science committee).

The institutional structure of the journal (whether it is associated with an academic society, a large publisher, both, or none) may also impact the creation and implementation of open science initiatives. Independent journals may find it easier to make changes to their policies as they do not need approval of publishers or scientific societies that sponsor the journals, but they may also have fewer financial resources for implementation. Some academic societies (for example the American Psychological Association: https://www.apa.org/pubs/journals/resources/open-science) and publishers (for example University of California Press) have encouraged open science policies and practices, and may offer support such as model policy language, ready-to-go widgets added to submission platforms, article templates with dedicated sections for disclosing open science practices, and access to paid platforms for data and code sharing. Journals affiliated with an academic society may have more hurdles to approve new initiatives compared to non-affiliated journals; however, being affiliated with a society might also make it harder to discontinue such initiatives, which is useful for making long-term change that is not limited only to the current editorial cohort. Indeed, editorial turnover can be a major barrier to not only maintaining the open science initiatives that were implemented, but also to ensuring that the initiatives are being carried out by people with sufficient background knowledge and motivation to oversee their effectiveness.

Changing funder requirements (for example Plan S from cOAlition S: https://www.coalition-s.org) and government regulations (for example the United States Office of Science and Technology Policy “Ensuring Free, Immediate, and Equitable Access to Federally Funded Research”) will have an impact on how editors may weigh the importance of adopting some of these initiatives. For example, with many funders requiring that outputs must be open access, editors may need to assess their journal’s current open access options if they wish to still remain a credible outlet for research in their field. In addition, technological innovations will make certain initiatives easier to adopt. For example, as cloud-based solutions for sharing data and code packages become the norm, the difficulty of reproducing analyses should decrease, which will in turn lessen the time and financial burden for a journal implementing pre-publication verification.

It is important to acknowledge that the technological landscape in publishing is ever-evolving. There will be topics we have not included that may become very important areas of social science journal editing in the near future. For example, the impact of large language models (LLMs, such as ChatGPT and Google’s Bard) on scholarly publishing is just beginning to be discussed [111–113], and yet ChatGPT has already been listed as an author on several research papers [114]. Cacciamani et al. are working together with academic and publishing regulatory organizations to develop guidelines for accountable reporting and use of LLMs in scientific research. Until more detailed guidelines are available, journals may emphasize that the use of LLMs should be declared on submission [111] and that ultimately authors are always responsible for the content of their submissions [113]. It is possible these tools could aid inclusivity by allowing researchers to easily improve the language used in their manuscripts before submission (a task that currently adds a large burden for non-native speakers of English, see [115]). It is also possible that LLMs could be used to facilitate the editing process (by, for example, highlighting key points that come up from multiple reviewers of the same paper). Future iterations of our full guide are likely to include a dedicated section on LLMs once further guidance is available.

## Conclusion

This guide provides an overview to help editors make informed decisions about the options available for increasing openness and transparency at their journal. In our discussion of increasing openness in science, we have focused on *journals* because this is a guide for *journal editors*. However, there are several other stakeholders who wield power to implement open science initiatives – including but not limited to – funders, research institutions, academic societies, and scholarly communication organizations such as publishers, preprint servers, and data repositories. Even sticking within scientific publishing, many other approaches may be impactful, ranging from incremental innovations within the current system to completely revolutionizing scientific knowledge dissemination. For example, some people argue for journals to play only a curatorial role, even without making accept or reject decisions [116]. Others argue that articles should be published and reviewed on preprint servers, and dedicated preprint peer review services should decide whether articles do or do not deserve to receive an endorsement or recommendation [117]. At the most “extreme” end of this spectrum, some argue that journals are unnecessary altogether (for example see *The Unjournal*: https://globalimpact.gitbook.io/the-unjournal-project-and-communication-space/).

Our guide keeps within the “incremental innovation” section of this spectrum, although some entries in the full guide are more disruptive to the current publishing model (for example breaking off from a traditional publisher and starting an independent journal). While advocating for a scientific knowledge dissemination revolution is beyond the scope of this article, we encourage readers to reflect on the scientific values underpinning our guidance and what an alternate scientific knowledge dissemination landscape could look like that is fully in line with these values. Editors should pursue options that make the most sense for their communities, taking into account the logistics of adopting different policies and practices, including the journal's scope, set up, and financial resources. Editors have an important role to play in the adoption of open science, in which they are supported by this abbreviated guide, the full guide [62], and the JEDI community.

## References

[1] Buckwalter W. The replication crisis and philosophy. PhiMiSci. 2022;3. Available from: https://philosophymindscience.org/index.php/phimisci/article/view/9193. Cited 2023 May 19.

[2] Button KS, Ioannidis JPA, Mokrysz C, Nosek BA, Flint J, Robinson ESJ (2013). Power failure: why small sample size undermines the reliability of neuroscience. Nat Rev Neurosci.

[3] Cook BG (2014). A Call for Examining Replication and Bias in Special Education Research. Remedial Special Educ.

[4] Farrar BG, Vernouillet A, Garcia-Pelegrin E, Legg E, Brecht K, Lambert P (2022). Reporting and interpreting non-significant results in animal cognition research.

[5] Ioannidis JPA (2012). Why Science Is Not Necessarily Self-Correcting. Perspect Psychol Sci.

[6] Ioannidis J, Doucouliagos C (2013). What’s to know about the credibility of empirical economics?: Scientific credibility of economics. J Econ Surv.

[7] Wright BE (2015). The Science of Public Administration: Problems, Presumptions, Progress, and Possibilities. Public Admin Rev.

[8] Smaldino PE, McElreath R (2016). The natural selection of bad science. Royal Society Open Science.

[9] Smaldino PE, Turner MA, Kallens PAC (2019). Open science and modified funding lotteries can impede the natural selection of bad science. Royal Soc Open Sci.

[10] Nosek BA, Spies JR, Motyl M (2012). Scientific Utopia: II. Restructuring Incentives and Practices to Promote Truth Over Publishability. Perspect Psychol Sci..

[11] UNESCO. UNESCO Recommendation on Open Science. 2021. Available from: https://unesdoc.unesco.org/ark:/48223/pf0000379949.locale=en. Accessed 12 Dec.

[12] Azevedo F, Parsons S, Micheli L, Strand J, Rinke EM, Guay S, et al. Introducing a Framework for Open and Reproducible Research Training (FORRT). OSF Preprints. 2019.

[13] Parsons S, Azevedo F, Elsherif MM, Guay S, Shahim ON, Govaart GH (2022). A community-sourced glossary of open scholarship terms. Nat Hum Behav.

[14] Nosek BA, Alter G, Banks GC, Borsboom D, Bowman SD, Breckler SJ (2015). Promoting an open research culture. Science.

[15] Levenstein MC, Lyle JA (2018). Data: Sharing Is Caring. Adv Methods Pract Psychol Sci.

[16] Nosek BA, Hardwicke TE, Moshontz H, Allard A, Corker KS, Dreber A, et al. Replicability, Robustness, and Reproducibility in Psychological Science. Annu Rev Psychol. 2022;73:719–48. 10.1146/annurev-psych-020821-114157 34665669

[17] Collins F, Morgan M, Patrinos A. The Human Genome Project: lessons from large-scale biology. (Viewpoint) (Special Section). Science. 2023;300(5617):286–90. 10.1126/science.1084564 12690187

[18] Errington TM, Denis A, Perfito N, Iorns E, Nosek BA (2021). Challenges for assessing replicability in preclinical cancer biology. eLife..

[19] Farrar BG, Voudouris K, Clayton N. Replications, Comparisons, Sampling and the Problem of Representativeness in Animal Cognition Research. PsyArXiv; 2020. Available from: https://osf.io/2vt4k. Cited 2023 May 19. 10.26451/abc.08.02.14.2021 PMC7610843 34046521

[20] Christensen G, Miguel E (2018). Transparency, Reproducibility, and the Credibility of Economics Research. J Econ Lit.

[21] Delios A, Clemente EG, Wu T, Tan H, Wang Y, Gordon M (2022). Examining the generalizability of research findings from archival data. Proc Natl Acad Sci USA.

[22] Miguel E, Camerer C, Casey K, Cohen J, Esterling KM, Gerber A (2014). Promoting Transparency in Social Science Research. Science.

[23] Tierney W, Hardy JH, Ebersole CR, Leavitt K, Viganola D, Clemente EG (2020). Creative destruction in science. Organ Behav Hum Decis Process.

[24] Tierney W, Hardy J, Ebersole CR, Viganola D, Clemente EG, Gordon M (2021). A creative destruction approach to replication: Implicit work and sex morality across cultures. J Exp Soc Psychol.

[25] Makel MC, Plucker JA (2014). Facts Are More Important Than Novelty: Replication in the Education Sciences. Educ Res.

[26] Cook BG, Lloyd JW, Mellor D, Nosek BA, Therrien WJ (2018). Promoting Open Science to Increase the Trustworthiness of Evidence in Special Education. Except Child.

[27] Gehlbach H, Robinson CD (2018). Mitigating Illusory Results through Preregistration in Education. J Res Educ Effect.

[28] McBee MT, Makel MC, Peters SJ, Matthews MS (2018). A Call for Open Science in Giftedness Research. Gifted Child Quarterly.

[29] Fleming JI, Wilson SE, Hart SA, Therrien WJ, Cook BG (2021). Open accessibility in education research: Enhancing the credibility, equity, impact, and efficiency of research. Educ Psychol.

[30] Lupia A, Elman C (2014). Openness in Political Science: Data Access and Research Transparency: Introduction. PS, Pol Sci Politics.

[31] Harris JK, Johnson KJ, Carothers BJ, Combs TB, Luke DA, Wang X (2018). Use of reproducible research practices in public health: A survey of public health analysts. Gilligan C, editor. PLoS ONE..

[32] Peng RD, Hicks SC (2021). Reproducible Research: A Retrospective. Annu Rev Public Health.

[33] Maienschein J, Parker JN, Laubichler M, Hackett EJ (2019). Data Management and Data Sharing in Science and Technology Studies. Sci Technol Human Values.

[34] Bornmann L, Guns R, Thelwall M, Wolfram D (2021). Which aspects of the Open Science agenda are most relevant to scientometric research and publishing? An opinion paper. Quant Sci Stud.

[35] Freese J (2007). Replication Standards for Quantitative Social Science: Why Not Sociology?. Sociol Methods Res.

[36] Freese J, King MM (2018). Institutionalizing Transparency. Socius.

[37] Rahal RM, Hamann H, Brohmer H, Pethig F (2022). Sharing the Recipe: Reproducibility and Replicability in Research Across Disciplines. RIO.

[38] Korbmacher M, Azevedo F, Pennington CR, Hartmann H, Pownall M, ..., et al. The replication crisis has led to positive structural, procedural, and community changes. MetaArXiv. 2023. 10.1038/s44271-023-00003-2 PMC11290608 39242883

[39] Michie S, van Stralen MM, West R (2011). The behaviour change wheel: A new method for characterising and designing behaviour change interventions. Implementation Sci.

[40] Atkins L, Francis J, Islam R, O’Connor D, Patey A, Ivers N (2017). A guide to using the Theoretical Domains Framework of behaviour change to investigate implementation problems. Implementation Sci.

[41] Norris E, O’Connor DB (2019). Science as behaviour: Using a behaviour change approach to increase uptake of open science. Psychol Health.

[42] Norris E, Munafo MR, Jay C, Baldwin J, Lautarescu A, ..., et al. Awareness of and engagement with Open Research behaviours: Development of the Brief Open Research Survey (BORS) with the UK Reproducibility Network. MetaArXiv. 2022.

[43] Naaman K, Grant S, Kianersi S, Supplee L, Henschel B, Mayo-Wilson E. Exploring enablers and barriers to implementing the Transparency and Openness Promotion (TOP) Guidelines: A theory-based survey of journal editors. MetaArXiv. 2022. 10.1098/rsos.221093 PMC9890101 36756061

[44] Evans TR, Pownall M, Collins E, Henderson EL, Pickering JS, O’Mahony A (2022). A network of change: united action on research integrity. BMC Res Notes.

[45] Stewart SLK, Pennington CR, Da Silva GR, Ballou N, Butler J, Dienes Z (2022). Reforms to improve reproducibility and quality must be coordinated across the research ecosystem: the view from the UKRN Local Network Leads. BMC Res Notes.

[46] Elman C, Kapiszewski D, Lupia A (2018). Transparent Social Inquiry: Implications for Political Science. Annu Rev Polit Sci.

[47] Aalbersberg I, Appleyard T, Brookhart S, Carpenter T, Clarke M, Curry S, et al. Making Science Transparent By Default; Introducing the TOP Statement. OSF Preprints. 2018.

[48] Mayo-Wilson E, Grant S, Supplee L, Kianersi S, Amin A, DeHaven A (2021). Evaluating implementation of the Transparency and Openness Promotion (TOP) guidelines: the TRUST process for rating journal policies, procedures, and practices. Res Integr Peer Rev.

[49] Grant S, Mayo-Wilson E, Kianersi S, Naaman K, Henschel B. Open Science Standards at Journals that Inform Evidence-Based Policy. Prev Sci. 2023;24:1275–91. 10.1007/s11121-023-01543-z 37178346

[50] Kathawalla UK, Silverstein P, Syed M (2021). Easing Into Open Science: A Guide for Graduate Students and Their Advisors. Collabra Psychol..

[51] Montoya AK, Krenzer WLD, Fossum JL (2021). Opening the Door to Registered Reports: Census of Journals Publishing Registered Reports (2013–2020). Collabra Psychol..

[52] TARG Meta-Research Group & Collaborators, Thibault RT, Clark R, Pedder H, van den Akker O, Westwood S, et al. Estimating the prevalence of discrepancies between study registrations and publications: A systematic review and meta-analyses. medRxiv. 2021. 10.1136/bmjopen-2023-076264 PMC10551944 37793922

[53] Chambers C, Dunn A. Rapidly reviewing Registered Reports: A retrospective. Blog posts and articles from the Royal Society. 2022. Available from: https://royalsociety.org/blog/2022/09/registered-reports/. Accessed 12 Dec.

[54] Scheel AM, Schijen MRMJ, Lakens D (2021). An Excess of Positive Results: Comparing the Standard Psychology Literature With Registered Reports. Adv Methods Pract Psychol Sci.

[55] Hummer L, Thorn FS, Nosek BA, Errington TM. Evaluating Registered Reports: A Naturalistic Comparative Study of Article Impact. OSF Preprints. 2017.

[56] Soderberg CK, Errington TM, Schiavone SR, Bottesini J, Thorn FS, Vazire S (2021). Initial evidence of research quality of registered reports compared with the standard publishing model. Nat Hum Behav.

[57] Chambers CD, Tzavella L (2022). The past, present and future of Registered Reports. Nat Hum Behav.

[58] Nosek BA, Lakens D (2014). Registered Reports: A Method to Increase the Credibility of Published Results. Social Psychology.

[59] Karhulahti VM (2022). Registered reports for qualitative research. Nat Hum Behav.

[60] Bergmann C. The Buffet Approach to Open Science. CogTales. 2023. Available from: https://cogtales.wordpress.com/2023/04/16/the-buffet-approach-to-open-science/. Accessed 12 Dec.

[61] Komssi M, Pichlis D, Raatikainen M, Kindstrom K, Jarvinen J (2015). What are Hackathons for?. IEEE Softw.

[62] Silverstein P, Elman C, Montoya AK, McGillivray B, Pennington CR, Harrison CH, et al. A Guide for Social Science Journal Editors on Easing into Open Science (FULL GUIDE). OSF Preprints. 2023. 10.1186/s41073-023-00141-5 PMC10870631 38360805

[63] Vazire S (2017). Quality Uncertainty Erodes Trust in Science. Collabra Psychol..

[64] Crüwell S, Apthorp D, Baker BJ, Colling L, Elson M, Geiger SJ (2023). What’s in a Badge? A Computational Reproducibility Investigation of the Open Data Badge Policy in One Issue of Psychological Science. Psychol Sci.

[65] Gabelica M, Bojčić R, Puljak L (2022). Many researchers were not compliant with their published data sharing statement: a mixed-methods study. J Clin Epidemiol.

[66] Stodden V, Seiler J, Ma Z (2018). An empirical analysis of journal policy effectiveness for computational reproducibility. Proc Natl Acad Sci USA.

[67] Rice DB, Moher D (2019). Curtailing the Use of *Preregistration*: A Misused Term. Perspect Psychol Sci.

[68] Kidwell MC, Lazarević LB, Baranski E, Hardwicke TE, Piechowski S, Falkenberg LS (2016). Badges to Acknowledge Open Practices: A Simple, Low-Cost, Effective Method for Increasing Transparency. Macleod MR, editor. PLoS Biol..

[69] Haven TL, Errington TM, Gleditsch KS, van Grootel L, Jacobs AM, Kern FG (2020). Preregistering Qualitative Research: A Delphi Study. Int J Qual Methods.

[70] Nosek BA, Ebersole CR, DeHaven AC, Mellor DT (2018). The preregistration revolution. Proc Natl Acad Sci.

[71] Pennington CR. A student’s guide to open science: Using the replication crisis to reform psychology. Maidenhead: Open University Press; 2023.

[72] Karhulahti VM, Backe HJ (2021). Transparency of peer review: a semi-structured interview study with chief editors from social sciences and humanities. Res Integr Peer Rev.

[73] Ross-Hellauer T, Horbach SPJM. ‘Conditional Acceptance’ (additional experiments required): A scoping review of recent evidence on key aspects of Open Peer Review. MetaArXiv. 2022.

[74] Vazire S. Implications of the Credibility Revolution for Productivity, Creativity, and Progress. Perspect Psychol Sci. 2018;13(4):411–7. 10.1177/1745691617751884 29961410

[75] Nosek BA, Errington TM (2020). What is replication?. PLoS Biol.

[76] Koole SL, Lakens D (2012). Rewarding Replications: A Sure and Simple Way to Improve Psychological Science. Perspect Psychol Sci.

[77] Wong PT (1981). Implicit editorial policies and the integrity of psychology as an empirical science. Am Psychol.

[78] Srivastava S. A Pottery Barn rule for scientific journals. The Hardest Science. 2012. Available from: https://thehardestscience.com/2012/09/27/a-pottery-barn-rule-for-scientific-journals/. Accessed 12 Dec.

[79] Vazire S, Holcombe AO. Where Are the Self-Correcting Mechanisms in Science? Rev Gen Psychol. 2021;26(2):212–23.

[80] COPE Council. COPE Retraction guidelines — English. 2019. Available from: 10.24318/cope.2019.1.4.

[81] Hardwicke TE, Thibault RT, Kosie JE, Tzavella L, Bendixen T, Handcock SA, et al. Post-publication critique at top-ranked journals across scientific disciplines: A cross-sectional assessment of policies and practice. Royal Soc Open Sci. 2022;9(8). 10.1098/rsos.220139 PMC9399707 36039285

[82] Moshontz H, Binion G, Walton H, Brown BT, Syed M (2021). A Guide to Posting and Managing Preprints. Adv Methods Pract Psychol Sci.

[83] Ortega RP. Science’s English dominance hinders diversity, but the community can work toward change. Science. 2020.

[84] Bahlai C, Bartlett LJ, Burgio KR, Fournier AMV, Keiser CN, Poisot T (2019). Open Science Isn’t Always Open to All Scientists. Am Sci.

[85] Bennett EA (2021). Open Science From a Qualitative, Feminist Perspective: Epistemological Dogmas and a Call for Critical Examination. Psychol Women Q.

[86] Elsherif M, Middleton S, Phan JM, Azevedo F, Iley B, ..., et al. Bridging Neurodiversity and Open Scholarship: How Shared Values Can Guide Best Practices for Research Integrity, Social Justice, and Principled Education. MetaArXiv. 2022.

[87] Puthillam A, Doble LJM, Santos JJID, Elsherif MM, Steltenpohl CN, Moreau D, et al. Guidelines to improve internationalization in the psychological sciences. Soc Pers Psychol Compass. 2023;e12847.

[88] Ross-Hellauer T. Open science, done wrong, will compound inequities. Nature. 2022;603(363):363. 10.1038/d41586-022-00724-0 35288691

[89] Whitaker K, Guest O. #bropenscience is broken science. The Psychologist. 2020. Available from: https://www.bps.org.uk/psychologist/bropenscience-broken-science. Accessed 12 Dec.

[90] Huber J, Inoua S, Kerschbamer R, König-Kersting C, Palan S, Smith VL. Nobel and novice: Author prominence affects peer review. University of Graz, School of Business, Economics and Social Sciences Working Paper. 2022. 10.1073/pnas.2205779119 PMC9564227 36194633

[91] Steltenpohl CN. To Sign or Not to Sign. 2020. Available from: https://cnsyoung.com/to-sign-or-not-to-sign/. Accessed 12 Dec.

[92] Tomkins A, Zhang M, Heavlin WD (2017). Reviewer bias in single- versus double-blind peer review. Proc Natl Acad Sci USA.

[93] Waltman L, Kaltenbrunner W, Pinfield S, Woods HB. How to improve scientific peer review: Four schools of thought. Learned Publishing. 2023;36:334–47. 10.1002/leap.1544. 10.1002/leap.1544 PMC10946616 38504796

[94] Boulton D, Hammersley M. Analysis of Unstructured Data. In: Data Collection and Analysis. 2nd ed. London: SAGE Publications Ltd; 2006. p. 243–59. Available from: 10.4135/9781849208802.

[95] Bennett A, Checkel JT (2014). Process Tracing: From Metaphor to Analytic Tool.

[96] Ragin CC (1987). The Comparative Method: Moving beyond Qualitative and Quantitative Strategies.

[97] Steltenpohl CN, Lustick H, Meyer MS, Lee LE, Stegenga SM, Standiford Reyes L, et al. Rethinking Transparency and Rigor from a Qualitative Open Science Perspective. JOTE. 2023. Available from: https://journal.trialanderror.org/pub/rethinking-transparency. Cited 2023 Jun 8.

[98] Adler JM, Singer JA (2023). Psychobiographies of social change agents: Introduction to the Special Issue. J Pers..

[99] Jacobs A (2020). Pre-registration and Results-Free Review in Observational and Qualitative Research. The Production of Knowledge: Enhancing Progress in Social Science.

[100] Jafar AJN (2018). What is positionality and should it be expressed in quantitative studies?. Emerg Med J..

[101] Jamieson MK, Govaart GH, Pownall M (2023). Reflexivity in quantitative research: A rationale and beginner’s guide. Soc Pers Psych.

[102] Makel MC, Plucker JA, Hegarty B (2012). Replications in Psychology Research: How Often Do They Really Occur?. Perspect Psychol Sci.

[103] Pownall M. Is replication possible for qualitative research? PsyArXiv. 2022.

[104] Devezer B, Nardin LG, Baumgaertner B, Buzbas EO (2019). Scientific discovery in a model-centric framework: Reproducibility, innovation, and epistemic diversity. Fanelli D, editor. PLoS ONE..

[105] DuBois JM, Strait M, Walsh H (2018). Is it time to share qualitative research data?. Qualitative Psychology.

[106] Jones K, Alexander SM, et al. Qualitative data sharing and re-use for socio-environmental systems research: A synthesis of opportunities, challenges, resources and approaches. SESYNC White Paper; 2018. Available from: 10.13016/M2WH2DG59.

[107] Tsai AC, Kohrt BA, Matthews LT, Betancourt TS, Lee JK, Papachristos AV (2016). Promises and pitfalls of data sharing in qualitative research. Soc Sci Med.

[108] Joel S, Eastwick PW, Finkel EJ (2018). Open Sharing of Data on Close Relationships and Other Sensitive Social Psychological Topics: Challenges, Tools, and Future Directions. Adv Methods Pract Psychol Sci.

[109] Casadevall A, Enquist L, Imperiale MJ, Keim P, Osterholm MT, Relman DA (2013). Redaction of Sensitive Data in the Publication of Dual Use Research of Concern. mBio..

[110] Christian TM, Gooch A, Vision T, Hull E (2020). Journal data policies: Exploring how the understanding of editors and authors corresponds to the policies themselves. Sugimoto CR, editor. PLoS ONE..

[111] Cacciamani GE, Collins GS, Gill IS. ChatGPT: standard reporting guidelines for responsible use. Nature. 2023;618(238). 10.1038/d41586-023-01853-w 37280286

[112] Hosseini M, Horbach SPJM (2023). Fighting reviewer fatigue or amplifying bias? Considerations and recommendations for use of ChatGPT and other large language models in scholarly peer review. Res Integr Peer Rev.

[113] Nature (2023). Tools such as ChatGPT threaten transparent science; here are our ground rules for their use. Nature..

[114] Stokel-Walker C (2023). ChatGPT listed as author on research papers: many scientists disapprove. Nature.

[115] Amano T, Ramírez-Castañeda V, Berdejo-Espinola V, Borokini I, Chowdhury S, Golivets M (2023). The manifold costs of being a non-native English speaker in science. Dirnagl U, editor. PLoS Biol..

[116] Eisen MB, Akhmanova A, Behrens TE, Diedrichsen J, Harper DM, Iordanova MD (2022). Peer review without gatekeeping. eLife.

[117] Avissar-Whiting M, Belliard F, Bertozzi SM, Brand A, Brown K, Clément-Stoneham G, et al. Advancing the culture of peer review with preprints. OSF Preprints. 2023.

